# *DNM1* mutation status, sex, and sterilization status of a cohort of Labrador retrievers with and without cranial cruciate ligament rupture

**DOI:** 10.1186/s40575-017-0041-9

**Published:** 2017-02-02

**Authors:** Kari J Ekenstedt, Katie M Minor, Aaron K Rendahl, Michael G Conzemius

**Affiliations:** 1 Veterinary and Biomedical Sciences Department, College of Veterinary Medicine, University of Minnesota, St. Paul, MN 55108 USA; 2 Veterinary Clinical Sciences Department, College of Veterinary Medicine, University of Minnesota, St. Paul, MN 55108 USA; 3 School of Statistics, University of Minnesota, Minneapolis, MN 55455 USA

**Keywords:** Exercise-induced collapse, Cranial cruciate ligament, Dog, Sex, Sterilization, Neuter, Spay

## Abstract

**Background:**

Exercise-induced collapse (EIC) due to *DNM1* mutation and rupture of the cranial cruciate ligament are both common syndromes in the Labrador retriever breed. A cohort of 313 Labradors was recruited based on their CCLR status and were subsequently genetically tested for EIC. Epidemiological aspects of the cohort were also described, including sex, sterilization status, and age at sterilization.

**Results:**

No sex difference was observed in dogs susceptible to EIC (homozygous for the mutant genotype) compared to dogs not susceptible to EIC (heterozygotes and dogs homozygous for the normal genotype). No evidence for association was detected between CCLR status and EIC status (*p* =0.357), although the sample cohort was not of sufficient size to entirely rule out an association. A significant difference (*p* = 0.031) was observed in the sex distribution of dogs affected with CCLR compared to those without CCLR. An increased number of female CCLR cases were observed compared to the number of female controls; male CCLR cases and controls were approximately the same number. When CCLR status was examined in each sex, no significant differences were observed between those that were sterilized and those that weren’t. However, for female dogs that were sterilized, CCLR cases were significantly higher in dogs sterilized at one year of age or younger compared to those sterilized when over the age of one year (*p* = 0.0021, OR 4.30, 95% CI 1.55–12.72); for males, this finding was suggestive, but not statistically significant (*p* = 0.0913, OR 3.57, 95% CI 0.809–14.476).

**Conclusions:**

CCLR is not associated with a large increase in EIC occurrence. Statistically, these two syndromes cannot be proven to be unrelated; however, concomitant occurrence of CCLR and EIC in Labrador retrievers is rare, despite the high prevalence of both syndromes in this breed. Epidemiological findings suggest that females may be over-represented in CCLR cases and that early sterilization (≤1 year) may increase the risk of Labradors developing CCLR later in life (particularly in females). These results should be considered preliminary and require confirmation in larger populations of Labrador retrievers.

## Plain english summary

Tear or rupture of the cranial cruciate ligament (CCL) in dogs is equivalent to humans tearing their anterior cruciate ligament (ACL), a knee ligament. CCL rupture (CCLR) in dogs can occur from trauma, but may also have genetic risk factors, because some breeds seem to be predisposed, including the Labrador Retriever. The definitive cause of CCLR has not been determined, but probably has many factors.

Labrador retrievers affected with exercise-induced collapse (EIC) have two defective copies of the *DNM1* gene. A genetic test can determine if a collapse episode is due to the *DNM1* mutation and predicts which dogs are susceptible to this form of collapse. During an EIC episode, a dog will usually first lose control of its hindlimbs, and may stumble while trying to continue running, all of which could stress the knee joint. Further, the *DNM1* mutation interferes with nerve signal transmission, which might impact the body’s ability to protect the integrity of joints, such as the knee. We wondered if EIC and CCLR, which are both common in the Labrador retriever breed, might be related. Therefore, we assembled a group of Labradors that were carefully screened to either be cases (have torn their CCL) or controls (normal knees) and subsequently genetically tested them for EIC. In addition, this was a good opportunity to describe the sex, sterilization status, and age-at-sterilization for this group of dogs.

Our results show that dogs with CCLR do not have EIC in a markedly higher frequency compared to dogs without CCLR. Therefore, if both syndromes are observed in the same dog, it’s probably just a coincidence due to how common both conditions are in the breed. From the epidemiological data, we observed a higher proportion of females in the CCLR cases compared to males. When we looked only at sterilized dogs, and divided them by what age they were sterilized, we saw a significantly higher proportion of CCLR cases in those dogs that were spayed or neutered early in life (before 1 year of age) in females. Ideally, these epidemiological findings should be confirmed in a larger population of dogs.

## Background

Exercise-induced collapse (EIC) and cranial cruciate ligament rupture (CCLR) are often observed in active Labrador retrievers. Dogs with EIC are normal when resting, but can manifest a ‘wobbly’ gait, which typically progresses to loss of control (flaccid paraparesis) of the hindlimbs, after a short duration of strenuous exercise [1]. The collapse episode typically resolves within 5–30 min [1]. This characteristic manifestation of collapse has an autosomal recessive mode of inheritance and has been strongly associated with a mutation in the dynamin 1 gene (*DNM1*) [2]. Depending on the sub-population of Labradors examined, the percentage of EIC homozygous mutant dogs varies from 1.8% (service Labradors - bred to assist humans with specific needs) to 13.6% (conformation Labradors - bred for showing), and the percentage of heterozygous (carrier) dogs varies from 17.9% (service Labradors) to 38.0% (field trial/hunt test Labradors) [3], indicating that this mutation and syndrome are common within this breed.

Cranial cruciate ligament rupture (CCLR) is a significant cause of pelvic limb lameness [4], with affected dogs displaying pain, instability of the joint, and altered kinematics [5]. A breed predisposition for CCLR has been reported in Labrador retrievers, with a 5.5-fold increased risk of developing CCLR [6]. Labrador retrievers and Labrador crosses are reported to account for 23% of all large breed CCLR cases (out of 228) in one study [7], and the Labrador retriever is reported as the most common breed (16% of cases, 70 out of 426 dogs) observed with CCLR in another study [8]. The definitive etiology of non-traumatic (i.e. idiopathic) CCLR in dogs is unknown, but it is likely multifactorial, and it may include genetic influences [9].

Dogs experiencing EIC-specific collapse lose coordination, often use a crouched posture (knee flexion) with their pelvic limbs [1], and may drag themselves by their forelimbs. According to Taylor et al., dogs with EIC often continue to run, dragging their crouched rear legs despite being in a collapse episode [10]. Taken together, these scenarios may subject the cranial cruciate ligament (CCL) to unknown additional mechanical stressors or damaging forces that could influence CCL integrity. Further, since the DNM1 protein, dynamin 1, functions in synaptic transmission, and genetically susceptible EIC dogs experiencing a collapse episode lose proper control of neurotransmission in the hindlimbs, this prevention of neurological input to the muscles may compromise the integrity of the CCL in affected dogs. The objective of this study was to determine if there is an association between these two common problems in the Labrador retriever breed by assembling a cohort of well-phenotyped CCLR cases and controls, which we would then genetically test for EIC, and from which we could also examine and report epidemiological aspects, such as sex and sterilization status.

## Methods

### Animals and phenotyping

North American dogs were recruited for this study as either CCLR cases or controls, with no consideration to sex or sterilization status, and were subsequently tested to determine their EIC genetic status. Therefore, no phenotype information was obtained regarding any dogs’ collapse status. After informed client consent was obtained, and each dog’s purebred Labrador retriever status was established (either by registration in or eligibility for registration in the American Kennel Club or the Canadian Kennel Club), case or control status was determined by stifle joint palpation by a board certified veterinary surgeon. The vast majority of CCLR cases (>90%) were defined by surgical confirmation of CCL tear, either unilateral or bilateral, with the remaining cases defined via physical exam, stifle palpation, and radiographs. Controls were defined as greater than 7 years of age [11] with no history of pelvic limb lameness or stifle surgery of any kind, and having no abnormalities in either knee via orthopedic examination (palpation only). Ages of case dogs ranged from 11 months to 15 years at the time of enrollment, although variable amounts of time may have elapsed post-injury. Information about each dog’s sex, sterilization status, and the age at which the dog was sterilized (divided into two categories: ≤ 1 year and > 1 year) was collected, however, all of these data were not available for every dog. When available, each dog’s age at sterilization, typically provided by the owner, was recorded. Dogs were recruited between 2010–2011 by the authors at the University of Minnesota’s referral hospital; a sub-set of samples were recruited during the same time frame by veterinary surgeons at the following specialty referral hospitals: Upstate Veterinary Specialties Clinic, in Latham, NY; Western College of Veterinary Medicine, in Saskatchewan, Canada, and Blue Pearl Referral Medicine, in Eden Prairie, MN. Controls were recruited contemporaneously with cases at all locations. The cohort consisted primarily of pet line Labradors.

### Genotyping

DNA was obtained either from whole blood or cheek swabs using standard extraction methods. Each DNA sample was calibrated to a standardized concentration and subjected to PCR amplification of *DNM1* exon 6, which contains the EIC mutant allele. PCR products were digested with restriction enzyme *SmlI* and visualized on 2% agarose gels as previously described [2]. EIC genotypes were categorized as either two copies of the mutant allele (homozygous E or EE), two copies of the normal allele (homozygous N or NN), or one copy of each allele (heterozygous/carrier, EN).

### Statistical analysis

The prevalence of EIC susceptibility in the entire cohort was calculated. Prevalence of CCLR in the Labrador breed could not be accurately calculated with this study due to ascertainment bias in recruiting for affected dogs. Descriptive statistics were performed for the distributions of sex for CCLR cases versus controls and for EIC genetic susceptibility; descriptive statistics were also performed for sterilization status and age at sterilization for CCLR cases versus controls. A Fisher’s exact test was used to test for association between sex and CCLR, sex and EIC, EIC and CCLR, sterilization status and CCLR for both sexes, and age at sterilization and CCLR for both sexes; odds ratios (OR) with a 95% confidence interval (CI) were calculated for each test. In addition, the test for the differences in proportions was used to compute the 95% confidence intervals on the EIC distribution in CCLR cases versus controls, and the Agresti-Coull method [12] was used to compute the confidence intervals on the proportion of sex distribution in CCLR cases versus controls. R (version 3.2.3) [13] was used to perform all statistical tests, and a *p*-value less than or equal to 0.05 was considered significant.

## Results

There were 313 Labrador retrievers recruited to the study; 174 were CCLR cases and 139 were CCLR controls (Table 1). Of the 174 CCLR cases, 71 were male and 103 were female, resulting in 59.2% females (95% CI: 51.8–66.2%). Of the 139 CCLR controls, 74 were male and 65 were female, resulting in 46.8% female (95% CI: 38.7–55.0%). In this cohort, the sex distribution of dogs with CCLR was significantly different (*p* = 0.031, OR 1.65, 95% CI 1.02–2.66) from the sex distribution of dogs without CCLR.

**Table 1:** CCLR case and control Labradors grouped by EIC genotype and sex

EIC Genotype and Sex							
	EE	EE	EN	EN	NN	NN	
CCLR Status	Male	Female	Male	Female	Male	Female	Total
Case	3	5	18	30	50	68	174
Control	1	2	24	19	49	44	139
Total	4	7	42	49	99	112	313

Based on their genotyping data, 11 dogs (3.5%) were susceptible to EIC (EE), 91 dogs (29.0%) were EIC carriers (EN), and 211 dogs (67.5%) were homozygous normal (NN). The sex distribution of dogs susceptible to EIC (7 females and 4 males) (Table 1) was not statistically different (*p* = 0.554, OR 1.53, 95% CI 0.38–7.28) from the sex distribution of dogs without EIC (EN + NN, 161 females and 141 males). This is consistent with an autosomal mutation; *DNM1* is located on canine chromosome 9 [2]. The allele frequency for the mutant (E) allele in this population was 18.05%.

2.5% of the dogs in the study had both CCLR and EIC. Possible association between CCLR and EIC was assessed using the Fisher’s exact test (Table 2). Of the 11 EIC-susceptible dogs, 8 were CCLR cases and 3 were CCLR controls. No statistically significant (*p* = 0.357) association was found between a diagnosis of CCLR and an EIC-susceptible genotype. However, the odds ratio was 2.18, with a 95% confidence interval of 0.51 to 13.0, indicating that the dataset does not allow definitive exclusion of association between these two conditions. Another way of looking at the data is to test for difference in the proportions: 4.6% of all CCLR cases had EIC-susceptible genotypes, while 2.2% of all CCLR controls had EIC-susceptible genotypes, a difference of 2.4%. The test for differences in proportions here gives a *p*-value of 0.3922 (95% CI -0.021–0.070).

**Table 2:** CCLR case and control Labrdors grouped by EIC phenotype

EIC Status			
CCLR Status	EE (Susceptible)	EN + NN (Non-susceptible)	Total
Case	8	166	174
Control	3	136	139
Total	11	302	313

Sterilization status was available for 311 of the 313 dogs (Table 3); since this was primarily a US pet population, unsurprisingly most of them (88.1%) were sterilized. Among females, 158 (94.6%) were spayed (96 CCLR cases and 62 controls) and 9 (5.4%) were intact (7 CCLR cases and 2 controls). The CCLR case distribution among females (spayed versus intact) was not statistically different (*p* = 0.4848, OR 2.25, 95% CI 0.41–22.89). Among males, 116 (80.6%) were neutered (61 CCLR cases and 55 controls) and 28 (19.4%) were intact (10 CCLR cases and 18 controls). The CCLR case distribution among males (neutered versus intact) was also not statistically different (*p* = 0.1409, OR 0.50, 95% CI 0.19–1.27).

**Table 3:** CCLR case and control Labradors grouped by sex and sterilization status

Sex and Sterilization Status					
	Female		Male		
CCLR Status	Intact	Spayed	Intact	Neutered	Total
Case	7	96	10	61	174
Control	2	62	18	55	137
Total	9	158	28	116	311

Statistical analyses were conducted separately for each sex

When accounting for a dog’s age at sterilization, differences between CCLR cases and controls in the female cohort became more apparent. Information regarding the dog’s age at sterilization was available for 185 dogs (Table 4). Among spayed female CCLR cases, 59 were spayed at or before one year of age, while 9 were spayed when older than one year of age. Among spayed female controls, 24 were spayed at ≤ 1 year of age, while 16 were spayed at > 1 year of age. This represented a significant difference (*p* = 0.0021, OR 4.30, 95% CI 1.55–12.72), with almost 87% of spayed female CCLR cases having been sterilized at or before one year of age. Among neutered male CCLR cases, 31 were neutered at or before one year, while only 4 were neutered when older than one year. Among neutered male controls, 30 were neutered at ≤ 1 year of age, while 12 were neutered at > 1 year of age. This was not statistically different (*p* = 0.0913, OR 3.05, 95% CI 0.809–14.476).

**Table 4:** Sterilized CCLR case and control Labradors grouped by sex and age at sterilization

Age at Sterilization					
	Spayed Female		Neutered Male		
CCLR Status	≤1 year	> 1 year	≤1 year	> 1 year	Total
Case	59	9	31	4	103
Control	24	16	30	12	82
Total	83	25	61	16	185

All dogs on this table are sterilized. Statistical analyses were conducted separately for each sex

## Discussion

Dogs homozygous for the EIC mutation were identified in 8 of 174 CCLR-affected Labradors (4.6%, 95% CI 2.21–8.95%) and 3 of 139 CCLR-normal Labradors (2.2%, 95% CI 0.46–6.44%), which is not a significant difference. Because the number of dogs affected with EIC in the overall pet population of Labradors is low, it is not surprising that we only detected a small number of dogs concomitantly affected with EIC susceptibility and CCLR. It was entirely unknown prior to this study how frequently dogs would be concomitantly affected with EIC and CCLR, therefore several hundred were recruited. Ultimately, though, this study was under-powered to draw strong conclusions about association of EIC and CCLR, due to the relative rarity of dogs with both conditions. This sample cohort demonstrates that CCLR and EIC-susceptibility might be unrelated (*p* = 0.357, Fisher’s exact test), and, if they are associated, that CCLR is not associated with large increases in EIC occurrence. The test for differences in the proportion of EIC-susceptibility in CCLR cases versus CCLR controls (a 2.4% difference was observed) tells us that this difference could go as much as 2% in one direction and 7% in the other direction, and this is not likely to be a clinically significant difference.

The overall EIC genotype distribution was 67.5% homozygous for the wild-type allele, 29.0% heterozygous, and 3.5% homozygous for the mutant allele (EIC-susceptible dogs). Because our entire cohort was recruited based on CCLR status, it is inappropriate to use this group to calculate prevalence of CCLR in the Labrador breed. However, since these dogs were only subsequently tested for EIC, and they were not recruited based on collapse phenotype, this represents an opportunity to examine EIC frequency in the breed. Our finding that 3.5% of 313 dogs were EE is consistent with the overall-breed frequency of 3% as previously reported [2], and it is also consistent with the reported percentage of 2.9% EE in “pet” Labrador lines [3]. The 29.0% carrier rate observed in the present study is also consistent with previously reported carrier frequencies: 37% [2] and 27.9% [3]. These results confirm that the mutant EIC allele is very common in the Labrador breed.

Actual collapse status of the 11 EIC-susceptible dogs is unknown due to the lack of follow-up investigation, which was not undertaken due to the post hoc nature of the EIC testing and the fact that penetrance of EIC-susceptible dogs actually experiencing collapse has been examined in other work. The EIC-associated mutation is not fully penetrant; previous work has shown that dogs homozygous for the EE allele and therefore collapse-susceptible can be phenotypically normal and not experience collapse. One study reported 9% of EE dogs had no history of collapse [2], while another reported that an average of 83.6% of EE Labradors had a collapse event by 4 years of age [3], indicating that an average of 16.4% of homozygous mutant dogs had not collapsed by that age. The authors suggested that this could be due to the dogs never having been exposed to sufficient exercise or excitement to trigger the collapse episode, or due to modifying genetic and/or environmental factors. Therefore, it is possible to speculate that not all 11 EE dogs in the present study experienced a collapse episode.

In our cohort, which was recruited without respect to sex or sterilization status, a sex distribution difference was observed between dogs with CCLR versus dogs without CCLR. However, while this was statistically different (*p* = 0.031, OR 1.65, 95% CI 1.03–2.66), it may not be clinically significant. With hundreds of dogs in the study, a more precise calculation of statistical significance is possible; this can allow detection of smaller (and clinically irrelevant) differences. The group of CCLR cases was 59% female while only 47% of the CCLR control dogs were female; given the confidence interval for these proportions (38.7% to 66.2%, using the Agresti-Coull method), there could be almost twice as many female CCLR cases as female CCLR controls in the global population of Labradors retrievers. This difference, if observed, likely would have clinical significance. However, one could argue that the confidence interval also includes the possibility of 50% case:50% control for both sexes. In effect, these findings can only suggest that female dogs may be predisposed to CCLR.

Some previous studies comprising multiple breeds of dog have not detected a sex effect on rupture of the cranial cruciate ligament. For example, Duval et al. did not detect a difference in prevalence of CCLR between females and males in a sample cohort of 201 dogs [6]. Guthrie et al. similarly reported that sex did not significantly affect the incidence of developing or presenting with bilateral CCLR in a report comprising 426 dogs representing 44 different breeds [8]. While both of these studies included sterilization status as a variable, and Duval et al. did detect an increased risk for CCLR in neutered males and females compared to intact males and females, respectively, neither of these studies recorded the dogs’ age at sterilization, which may have resulted in different findings. Conversely, in a 2011 study by Adams et al., females were found to be twice as likely to suffer CCLR compared to males (189 cases, multiple breeds) [14]. Very few studies have focused exclusively on Labradors with CCLR. One study looking specifically at Labrador retrievers determined that sex did not affect likelihood or rate of rupturing the contralateral CCL after tearing the first CCL, or presenting initially with bilateral CCL ruptures, however this study only represented 94 Labradors and had no CCLR-normal dogs [15]. A Labrador-specific study by Hart et al. that included CCLR cases and controls reported 2.4% of males (19/801) were CCLR-affected and 2.5% of females (17/681) were CCLR-affected [16].

Taken together, it is unclear whether the statistically significant difference in sex distribution of CCLR-cases versus CCLR-controls in the present study is clinically significant or not, particularly since previous work offers mixed results and many previous studies did not consider age at sterilization as a variable. The question of sex differences for CCLR deserves further investigation. As a final perspective, it is interesting to note that the rate of anterior cruciate ligament (ACL) rupture in humans is three times higher in females compared to males [17]. There are likely multiple reasons for this sex difference in humans.

Human orthopedic disease studies typically do not need to consider the effects of lost gonadal hormones (at least in premenopausal women), whereas the majority of US dogs are sterilized, often at a young age, and there has been concern that the early loss of gonadal hormones may influence orthopedic disease development later in life. The removal of gonadal hormones can delay long-bone growth plate closure [18, 19], which may result in stifle joint angle or other changes that predispose the dog to CCLR. One study examining CCLR across several breeds found that sterilized dogs of either gender were significantly more likely than sexually intact dogs to have CCLR [20]. The present study had sterilization status information for all but two dogs and counters these findings, as no statistically significant differences were observed between dogs that were intact versus those that were sterilized when examining CCLR frequency separately amongst females and males (Females: *p* = 0.485, OR 2.25, 95% CI 0.41—22.89; Males: *p* = 0.141, OR 0.503, 95% CI 0.19—1.27). A more balanced population of intact dogs to sterilized dogs would allow drawing of stronger conclusions; as it stands, the confidence intervals, particularly with the females, are wide.

Since there were so few intact dogs in the present study, the subset of all sterilized dogs were examined for the age at which they sterilized, and whether this was associated with CCLR status. Differences were observed between those sterilized at a young age (≤ 1 year) versus an older age (>1 year). For both sexes, more CCLR cases were observed in dogs sterilized at a young age compared to those sterilized at an older age, and these differences were statistically significant for the females, though they were not quite statistically significant in the males.

Other studies have also examined the age at sterilization of CCLR cases. In one study, which looked exclusively at Golden Retrievers, a significantly higher percentage of both male and female early-neutered (sterilized at < 12 months) dogs had CCLR compared to intact dogs and to late-neutered (≥ 12 months) dogs [21]. Another study, which examined exclusively German Shepherds, found a significantly higher occurrence of CCLR in both males and females that were neutered at less than one year of age compared to intact dogs of the same sex [22]. A Labrador retriever-specific study found that males neutered at ages < 6 months had significantly more CCLR than intact males and that CCLR was increased in females with early sterilization, but not at a significant level [16]. These studies, with the exception of the Golden Retriever study [21], make statistical comparisons between a neutered-young group and the intact group, while the present study is comparing the frequency of CCLR cases exclusively in sterilized dogs (less than one year versus over one year at sterilization); therefore not all results are entirely comparable.

While it is tempting to simply conclude that sterilization at a younger age predisposes a dog to CCLR, and the present cohort supports such a conclusion, it should not be ignored that there were many CCLR controls, both male and female, that were similarly neutered at a young age. Also, it is important to note that in this study typically the owner, and not a medical record, provided the age at the time of their pet’s sterilization, introducing a potential inaccuracy. In addition, if there is a relationship between age of sterilization and CCLR occurrence, there is no current biologic explanation for a dichotomous (e.g., < or > 12 months of age) relationship. It is difficult to draw firm conclusions regarding what impact sterilization, at any age, has on development of CCLR with only this cohort, but, taken together with results from previous studies, these data suggest that early sterilization might be a risk factor for eventual development of CCLR. Prospective evaluation of this association would address potential errors or bias in data collection and allow for a more precise evaluation of the relationship.

The lack of follow-up information on these dogs is a limitation of this study. Without lifetime follow-up, it is possible that a subset of any of the controls (sterilized or intact) may have developed CCLR at a time after their enrollment in the study. Additionally, a subjective body condition score (BCS) was not consistently or uniformly assessed in this cohort of dogs. Excess weight has been suggested to contribute to CCLR [6, 14], but does not appear to be predictive for whether or not a Labrador will rupture the contralateral CCL after tearing the first CCL [15]. Certainly carrying excess weight is assumed to add to the biomechanical stresses on a joint.

Finally, given the size of the present cohort (~300 dogs, and for the age at sterilization data <200 dogs), these results should be considered preliminary, and in need of further confirmation in larger populations. Such limitations deserve to be addressed in future work attempting to better understand the risks and causes of CCLR in Labrador retrievers, which hopefully will shed further light on what risk, if any, is truly contributed by EIC status, sex, sterilization status, and age at sterilization.

## Conclusions

We did not identify a significant association between a diagnosis of CCLR and having the EIC-susceptible genotype. There are reasons other than *DNM1* mutation homozygosity which might cause a dog to collapse during exercise [23] any of which, along with an EIC collapse episode, could still contribute to a random individual dog tearing its CCL. But, our study was ultimately underpowered to definitively exclude association between EIC and CCLR; instead, we demonstrate that, even if the two syndromes are related, CCLR is not associated with a clinically significant increase in EIC. The high prevalence of both EIC and CCLR in the Labrador retriever population has likely resulted in the coincidental occurrence of both diseases in some individuals. Our cohort of Labradors also provided interesting epidemiological results in terms of sex distribution, sterilization status, and age at sterilization. Results suggest that females may be over-represented in the CCLR case category compared to males, and that sterilization *per se* is not a risk factor for development of CCLR, while early age at sterilization may increase a dog’s risk of developing CCLR, particularly for females. The finer points of these epidemiological aspects are beyond the scope of this study and deserve further investigation, with the addition of BCS data and even genetic data, in light of the multifactorial nature of CCLR.

## Abbreviations

ACL: Anterior cruciate ligament
BCS: Body condition score
CCL: Cranial cruciate ligament
CCLR: Cranial cruciate ligament rupture
CI: Confidence interval
EIC: Exercise-induced collapse
OR: Odds ratio
